# Methods for the Modification and Evaluation of Cereal Proteins for the Substitution of Wheat Gluten in Dough Systems

**DOI:** 10.3390/foods10010118

**Published:** 2021-01-08

**Authors:** Javier Espinoza-Herrera, Luz María Martínez, Sergio O. Serna-Saldívar, Cristina Chuck-Hernández

**Affiliations:** Tecnologico de Monterrey, School of Engineering and Sciences, Ave. Eugenio Garza Sada 2501, Monterrey 64849, Mexico; A00820228@itesm.mx (J.E.-H.); luzvidea@tec.mx (L.M.M.); sserna@tec.mx (S.O.S.-S.)

**Keywords:** gluten, prolamin, sorghum, maize, rice, protein modification

## Abstract

The substitution of wheat gluten in the food industry is a relevant research area because the only known treatment for celiac disease is abstinence from this protein complex. The use of gluten-free cereals in dough systems has demonstrated that the viscoelastic properties of gluten cannot be achieved without the modification of the protein fraction. The quality of the final product is determined by the ability of the modification to form a matrix similar to that of gluten and to reach this, different methods have been proposed and tested. These procedures can be classified into four main types: chemical, enzymatic, physical, and genetic. This article provides a comprehensive review of the most recent research done in protein modification of cereal and pseudocereals for gluten substitution. The reported effects and methodologies for studying the changes made with each type of modification are described; also, some opportunity areas for future works regarding the study of the effect of protein modifications on gluten-free products are presented.

## 1. Introduction

**Figure 1:**
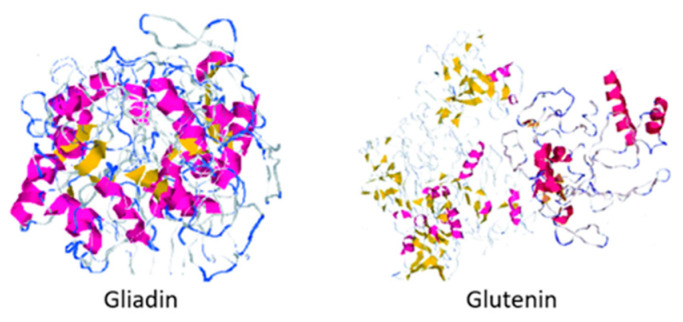
Protein structure of gliadin and glutenin. Figure edited from Rasheed et al. [10].

Approximately one out of one hundred people suffer from some celiac disease symptoms, an enteropathy caused by the consumption of specific peptide sequences in dietary gluten by people sensitive to its protein composition and whose only treatment is the absence of the protein from the diet [1,2,3]. Gluten is the nitrogen storage protein matrix found in wheat and cereals belonging to the Triticeae tribe, and it is composed of two proteins: gliadin and glutenin (shown in Figure 1) [4,5]. This protein complex is essential during the breadmaking process since the gluten matrix is responsible for retaining carbon dioxide during fermentation and the final volume of the product [6,7]. The global market size for gluten-free products during 2019 was estimated at USD 21.6 billion and is expected to increase at a compound annual growth rate (CAGR) of 9.2% from 2020 to 2027 [8]. Because of this increase in demand and the rising prevalence of celiac disease and Irritable Bowel Syndrome (IBS), the use of substitutes for the preparation of gluten-free baking products has been studied [8,9].

For a product to be considered gluten-free, the protein concentration must not exceed the value established by the corresponding government regulatory agencies; if this value is not low enough, it is considered a very-low gluten product containing a maximum of 100 mg/kg [11,12]. A product can be labeled gluten-free in the United States and Europe if it contains less than 20 mg/kg of gluten [13,14]. In other countries such as Mexico, a gluten-free product can be labeled as such, as long as the protein complex is removed and the total content of nitrogen does not exceed 500 mg/kg, expressed in dry matter [15]. Because of this low or null concentration of gluten, gluten-free bread cannot reproduce the properties seen in its unsubstituted counterparts, which translates to lower quality products.

Like maize, sorghum, and rice, gluten-free cereals do not form a viscoelastic gluten matrix and thus cannot entrap gas during the breadmaking process, producing low-quality products. The nutritional quality of cereal’s prolamins is considered low due to the lack of lysine and poor protein content (9–16% compared to 17–22% in gluten products) [16,17]. To make up for the absence of gluten matrix, the use of additives on gluten-free cereal doughs (formulation based on starch) and the modification of its protein content have been tested to create higher quality foods [6,18,19]. While both methods have yielded positive results, protein modifications can be preferred because of their stronger networks, barrier properties, and higher nutritional value than carbohydrate sources, like in the case of starch-based formulations [20,21]. These protein modifications have been classified as chemical, enzymatic, thermo-mechanical (also called physical), and in some cases, even genetic [22]. These classifications are defined and explained in Section 3 of this review. Studies focusing on proteins extracted from non-cereal sources have been made, such as in the case of β-conglycinin from soybeans; however, because this review focuses on cereal-based proteins, they will not be mentioned [23].

The potential that a gluten-free formulation has after modifying its protein content can be understood by studying the rheological behavior (strength and elasticity), thermal, and textural properties of the product [24]. These functional properties are affected by the stability and behavior of the protein content, which is why modifications can be made mainly to optimize dough handling properties, bread volume, and internal crumb texture [25]. Gluten-free cereals present lower functional values, especially in dough rheology compared to wheat gluten dough, which is why modifications focus on improving protein functionality. Recent developments in modifying proteins from gluten-free cereals and the methods used to study their potential as a gluten substitute are discussed in this review.

## 2. Cereals and Pseudocereals as the Protein Source in Bread-Making

The breadmaking process is commonly divided into three steps: mixing, fermentation, and baking [6,26]. As the name suggests, the mixing process is the step where all the ingredients for the dough are incorporated in order to develop a protein network; this is achieved by the kneading and hydration of gliadin and glutelin, which will form the continuous gluten network via covalent (disulfide bonds), non-covalent (hydrophobic), and hydrogen bonding interactions [27,28,29]. The dough mixing step is critically important because the mechanical work should be stopped when the gluten achieves its maximum strength, under or overmixed dough commonly yield low-quality bread, especially in terms of bread volume and crumb texture [30]. During the fermentation process, the yeast added in the mixing phase will ferment glucose, fructose, maltose, sucrose, and maltotriose, producing carbon dioxide (CO_2_) and ethanol; the CO_2_ will be trapped and retained by the gluten matrix, making the dough rise [4,7,31]. In gluten-free doughs, the replacement of gliadin with another prolamin would prevent the formation of the elastic network diminishing dough expansion. The final step in the breadmaking process will further enhance the gas expansion until cell rupture, thanks to the higher gas pressure induced by the increase in temperature, giving way to the bread’s final volume and appearance [32].

Wheat gluten is needed to achieve some final properties of dough and bread, such as viscosity, extensibility, and elasticity [33]. Therefore, the formulation of gluten-free bread is challenging since the protein content of other cereal sources do not exert these relevant rheological dough properties, resulting in products with insufficient rheological and baking properties [30]. Instead of presenting gliadin in their structure, gluten-free cereals contain other prolamins, which are the family of proteins to which gluten belongs.They are alcohol soluble storage proteins found in the endosperm of cereals and specific to different types of cereals but do not produce (after digestion) the *alpha*-gliadin peptide composed of 33 amino acids related to the celiac disease [9].

**Figure 2:**
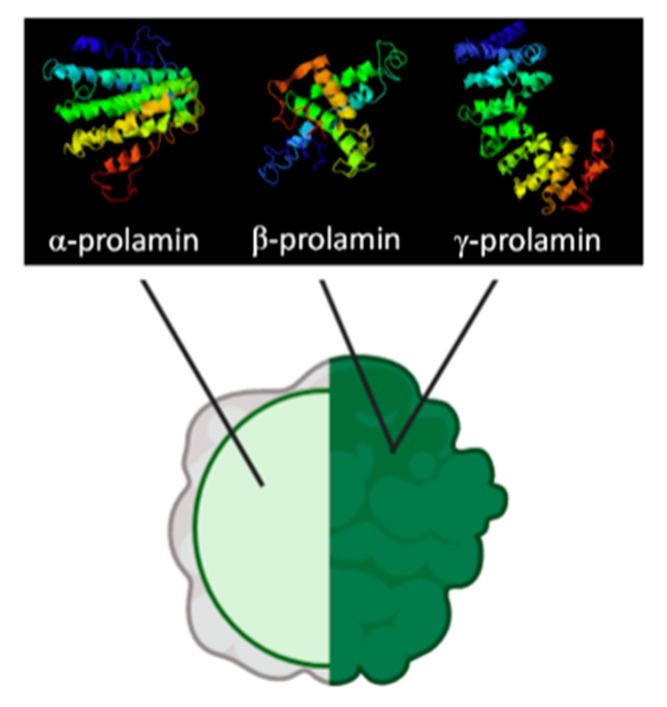
Composition of encapsulated prolamin bodies presented in maize and sorghum. Adapted from de-Mesa et al. and Dianda et al. [22,39].

Prolamins from gluten-free cereals have been used in breadmaking processes with varying results; however, the consensus is that they yield poor quality bread, with low specific volume, crumb softness, and higher staling rates [34]. Zein, the maize prolamin, has been one of the most widely studied proteins for the substitution of gluten and can be classified into α, β, γ, and δ fractions, of which approximately 70% of the protein content represents α-zein, a hydrophobic fraction rich in glutamine residues that makes corn flours challenging to hydrate [35,36,37]. Another factor that contributes to the poor performance of zein is that, unlike other proteins in cereals, which are present in the matrix, zein is encapsulated in protein bodies (see Figure 2); this makes it unable to interact with other components in the dough during mixing and will not create a network for gas retention and expansion [38].

Another prolamin studied for gluten substitution has been kafirin from sorghum, which shares some similarities with maize prolamins. Like zeins, kafirins are located inside of spherical protein bodies surrounded by starch granules in the glutelin matrix, which, as discussed above, limit their network forming capabilities [22,40,41]. Other similarities include their classification into α, β, γ, and δ fractions, and the predominance of α fraction (80–84% of prolamin), which is also rich in glutamine and non-polar amino acids (alanine, leucine, and proline) [40,42]. Compared to zeins, kafirins are more hydrophobic (12.5% increase in hydration energy), a quality bestowed by the extensive disulfide bonding of cysteine residues in its composition; providing water vapor and gas retention properties to films made from this protein but also bringing unwanted rigidity and stiffness to the product [38,40,42,43].

**Figure 3:**
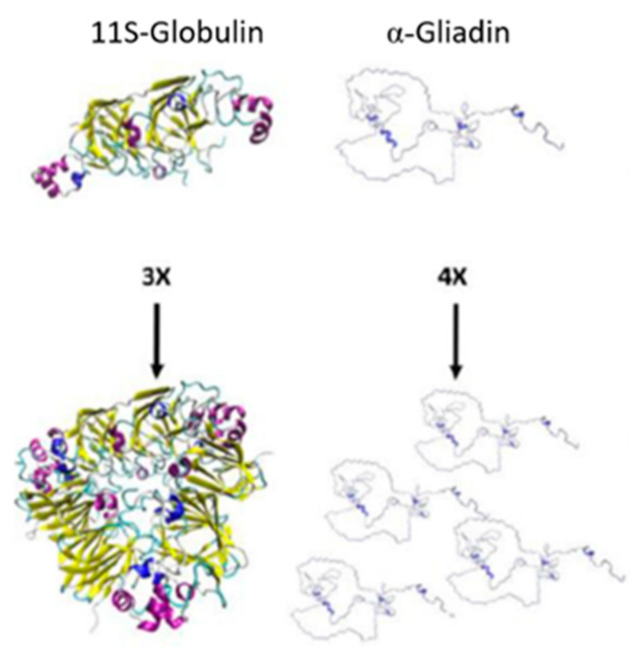
Subunits and polymers of 11S-globulin in pea and α-gliadin in wheat. 3× and 4× indicate the number of subunits shown. Adapted from Rasheed et al. [47].

Prolamins are not the only major storage proteins found in the endosperm of cereals. In rice, its storage protein content is mainly constituted of globulins and not its prolamin, orizein [9,44]. Globulins are protein fractions soluble in salt solutions and present a more globular structure than prolamins (Figure 3), where the globulin subunits comparatively form a more spherical shape [45,46,47]. Unlike zein and kafirin, rice proteins have not been classified in the same fractions; they are distinguished by their molecular weight and attributed to the globulins 11–12S families representing 70–80% of its total protein content in their endosperm, which is rich in glutamic acid, aspartic acid and arginine (non-essential amino acids) [48,49]. Studies for rice have shown that the degradation of the protein matrix of rice is beneficial for the formation of gluten-free doughs with no detrimental effects; this is because starch is the main structural component of gluten-free bread and the absence of protein gives it improved continuity and better batter properties [6,50].

Most pseudocereals used in breadmaking are dicots, and like rice, they are rich in starch and have protein constitutions predominated by globulins [51,52]. Compared to cereals, pseudocereals have larger protein-rich quantities in arginine, tryptophan, lysine, and histidine, and 7S and 11S globulins in their constitution [53]. Due to their higher protein quantity compared to cereals, pseudocereals such as amaranth, quinoa, and buckwheat have been used in gluten-free bread to improve shelf-life by adding their flours to the dough [54]. Table 1 summarizes the protein composition of cereals and pseudocereals commonly used in gluten substitution studies. These sources lack gliadin as a storage protein, and the major non-polar compositions for these grains have hindered their inclusion in bread formulations.

## 3. Protein Modification in Gluten-Free Dough

To achieve similar properties to gluten or improve the quality of the final product, protein modifications have been studied, and the most recent works are presented in this section. These modifications have previously been classified into three categories: chemical, enzymatic, and thermo-mechanical, the former also referred to as physical modifications herein [22]. This section also includes a fourth group: genetic modifications, because even though they are not applied directly to flours, the modification of DNA has effects on the final products’ protein composition. A summary of recent works on protein modification for gluten substitution is shown in Table 2, showing that maize, sorghum, and rice proteins have been the most extensively studied recently.

### 3.1. Chemical Modifications

These modifications are referred to as the use of chemical reagents, ingredients, or additives to induce changes in protein structure via bonds or conformational modifications; this can also include extraction methods where the final structure of the protein is changed [73,74]. As long as the extraction solvents modify the protein content of the cereal, they can be classified as a chemical modification; for the case of prolamins: ethanol and glacial acetic acid have been successfully used [75].

One example of a chemical modification performed with extraction solvents is documented by Taylor et al. They extracted the γ-kafirin fraction from sorghum using sodium lactate containing 2-mercaptoethanol to study the effects of the absence of this fraction on the rheology of resins [63]. Although the resins’ firmness was improved by removing γ-kafirin, after 16 days of storage, it lost its elasticity (36% stress recovery) and became a viscous resin, increasing its maximum force by 580%, surpassing the 6.9 N needed for gluten-containing resins. The removal of the γ fraction was expected to decrease the stiffening of resins because of its high amount of cysteine disulfide bonds; instead, the abundance of large chain β-kafirin increased the viscosity of the resin, eliminating its elasticity and proving that the presence of γ-kafirin is necessary to maintain a viscoelastic behavior [38,76].

Another type of chemical modification used to improve dough properties is the use of hydrocolloids, water-soluble polysaccharides that control an aqueous system’s rheology and texture when hydrated, forming hydrogen bonds or structures that stabilize the system [18,77]. In Raungrusmee et al.’s work, a protein-hydrocolloid non-covalent interaction formed in the modification induced a conformational change in the protein. In their work, xanthan gum and inulin were added to rice flour to formulate gluten-free noodles to improve their physical properties [68]. Both hydrocolloids produced a smoother and more consistent protein-hydrocolloid matrix when rice bran was added, which resulted in an increase in firmness from 0.08 ± 0.50 to 1.59 ± 0.14 N, as well as giving the noodles tensile strength (0.21 ± 0.04 N) and elasticity (14.03 ± 1.82 mm), properties that could not be measured without the modification. The addition of rice bran as an external source of protein favored the formation of hydrogen bonds between the added components and, similarly to the previous chemical example, increased the firmness of the noodles. These two examples indicate that the composition and configuration of proteins play a significant role in chemical modifications.

### 3.2. Enzymatic Modifications

Enzymatic modifications consist of enzymes’ use to enhance protein changes that, in turn, enhance their functionality in bakery systems. This type of modifications shows many advantages over chemical modifications, mainly higher reaction rates (up to 10^17^ times), specificity, and safer (less toxic) reaction conditions, which is why they have been investigated to further improve bread performance by enhancing protein crosslinking to form a better protein network or by hydrolyzing bonds to change protein structure and increase interactions [22,78,79]. Previously studied enzymes to modify gluten-free baking products include proteases, transglutaminases (TGs), oxidases, and amylases; however, the last two cannot be considered protein modifications since they work with non-protein components of doughs. More specifically, glucose oxidase oxidizes the hydroxyl groups of starch molecules, whereas amylase hydrolyzes the same complex polysaccharide into simpler forms, mainly dextrins [50,80,81].

**Figure 4:**
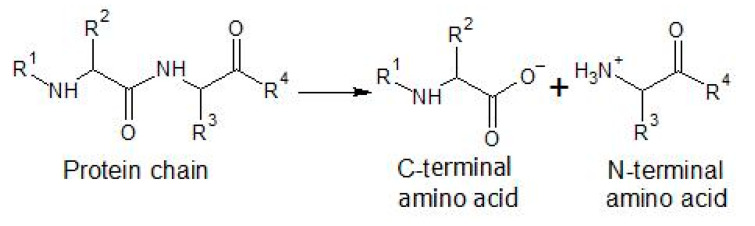
Hydrolyzation of the peptide bond present in amino acids.

Proteases are degradative enzymes that hydrolyze the peptide bond (shown in Figure 4) present in polypeptide chains and used in protein modification because of their specificity and selectivity [82]. Honda et al. studied the effect of several proteases on the rheological properties of gluten-free rice bread [67]. In their work, Papain, Protin SD-AY, and Protin SD-NY increased the specific volume by 33%, 19%, and 63%, respectively, and all three enzymes raised the elastic moduli 5.6 times and the viscous moduli 3.5 times, showing more elastic doughs that promoted volume expansion. The only protease that did not show this behavior was Newlase F, an enzyme unable to form small protein aggregates, needed to assist the protein network building, decreasing both the specific volume and viscoelastic properties.

In another study by Azizi et al., protease, and lipases were studied in conjunction with quinoa flour on rice bread quality [66]. Like the previous work by Honda, the addition of enzymes showed increased specific volume (4.3% regarding control), with 15% of rice flour substitution with quinoa flour. This further demonstrated the positive effect of protease on specific volumes, indicating that the protein interactions between the hydrolyzed chains can increase the dough’s capacity to retain gas. It is important to note that in Azizi’s work, the specific volume increase was lower, resulting from the additives used in their formulation, indicating that non-protein content can hinder the effect of the enzyme.

**Figure 5:**
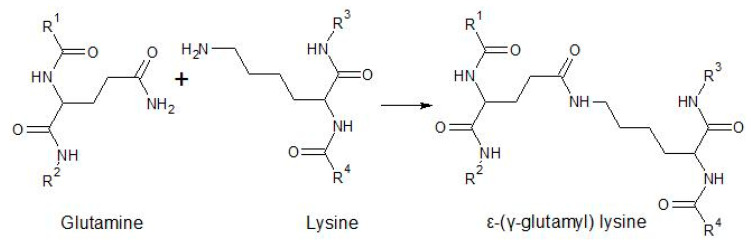
Formation of the ε-(γ-glutamyl) lysine bond between glutamine and lysine catalyzed by transglutaminase (TG).

Another enzyme studied in gluten-free cereals is transglutaminase, a transferase that catalyzes the formation of iso-peptide bonds including ε-(γ-glutamyl) lysine, a bond formed between the γ-carboxamide group in glutamine and the ε-amine of lysine (acyl acceptor), as shown in Figure 5 [83,84]. Although it is not a study of a gluten-free system, Tunçil et al. used TG in a wheat formulation containing highly digestible high lysine (HDHL) sorghum, a genotype with an altered folded protein body that makes kafirin more accessible to digestible enzymes [70]. TG addition increased the bread firmness up to 50% in doughs containing 20 and 30% sorghum substitution; however, the specific volume was not affected by the treatment. It has been proven that zein has insufficient lysine residues to carry out the crosslinking reaction, and because kafirin contains low lysine (0.1%), the same inefficiency can be expected [40,85]. Considering this finding, the prolamins of these high lysine genotypes may not contain enough of this basic amino acid residue for reaction with TG, making this enzyme ineffective for modifications.

### 3.3. Physical Modifications

The modification of a protein by thermal or mechanical external sources is referred in this review as physical modifications. In these investigations, flours obtained from traditional methods are subjected to physical treatments, such as heating, freezing, and extrusion, to modify the protein structure and functionality [25,86]. These types of modifications have been of interest to the food industry in recent years since no chemical reagents are needed during processing favoring green labeling, making it the chief reason why most gluten-free modifications have been produced with products processed by physical means [87]. Physical modifications have previously been categorized as thermo-mechanical in other references, and this denomination can still be used to differentiate the types of changes: thermal (heat treatments and microwave radiation) and mechanical (extrusion, microfluidization, and ultrasonication).

Thermal treatments have been studied as physical modifications because of the ability of high temperature to change the structure and stability of proteins; they can encompass simple heat treatments and processes where the resulting forces cause a change in temperature [88,89]. Marston et al. used heat treatment on sorghum flours to show that by increasing the treatment time, the specific volume and peak viscosity can be improved up to 17.6% and 2.4%, respectively [72]. These effects were attributed to the oxidation of free sulfhydryl groups in cysteine, increasing the amount of disulfide crosslinking, which yielded stronger doughs and larger loaf volumes. This study shows that by increasing the temperature of doughs, the protein content can be sufficiently altered to create interactions that favored gas retention.

Microwave radiation is classified as a thermal treatment since microwaves enhance agitation of water molecules, increasing thermal energy, and denature proteins by modifying mainly through unfolding [90]. In a study by Villanueva et al., microwave treatment on rice flour improved the specific volume of the bread from 3.3 mL/g to 4.6 mL/g and showed an increase in both elastic and viscous modules by 135% and 78%, respectively [69]. The study shows that even though the microwave treatment decreased up to 60.5% the critical dough firmness parameter, this method can still help diminish or prevent bread staling rate, which was reduced by up to 70% thanks to the improved protein interactions.

The shear and friction achieved inside an extruder increase internal temperatures of food materials [91]. Federici et al. found that when thermo extruding zein mixed with rice starch at 160 °C, the elasticity of the protein improved 23.8% due to increased prolamin molecular weight [64]. This temperature coincides with the glass transition of zein, where its structure is modified to a more denatured state, facilitating disulfide bonding and the formation of higher molecular weight protein [64,92]. The study concludes that the small size of the rice starch granules helped in the creation of a more continuous protein matrix, producing doughs with higher peak stress (0.56 ± 0.11 MPa) when compared to maize (0.39 ± 0.08 MPa) and potato starch (0.24 ± 0.09 MPa), both of which produce larger starch granules. This study can also be considered a mechanical treatment since shearing during extrusion can produce new non-covalent interactions between proteins and other dough components [93].

Mechanical treatments work by changing the particles’ size, creating new interactions between the dough elements. In addition to extrusion, the microfluidization and ultrasonication have been alternatively employed to enhance mechanical changes. The use of microfluidization during milling was used by Ozturk et al. to disintegrate the hydrophobic nature of zein, improving its properties by particle size reduction [65]. Their findings show that this technology lowers the specific volume of the final bread, which they solved by adding HPMC and guar gum as hydrocolloids, increasing the volume by 61.7% and 12.1% correspondingly. The authors attributed the decrease of specific volume and number of crumb pores or loci to the particles’ size obtained from the process. Unfortunately, the elastic properties enhanced during microfluidization were not enough to retain gas as strongly; in a previous study of zein particle size, the 100–140 μm range was shown to maximize the resistance and extensibility of the dough, making these sizes desirables when working with this protein as gluten substitute [94].

Likewise, ultrasonication is known to decrease the molecular weight and size of vegetable proteins; however, some cereal grains like rice and wheat have reported no changes after being exposed to ultrasound frequencies [95,96,97]. This behavior shows that the effectiveness of the modification would depend on the origin and physical properties of the protein, more specifically related to its mechanical strength [96]. Sullivan et al. showed that this technique could decrease the molecular weight of kafirins when using a frequency of 20 kHz and stated a 14.7% increase in small molecular weight proteins (0.075–0.5 kDa) observed [98]. Although the smaller protein size can make the technique attractive for protein modification, Sullivan’s study also mentions that the ultrasonication method increases the random coiling of secondary structures of kafirins, which is related to the loss of structure stability [99]. Nevertheless, no studies have been made on the breadmaking capabilities and properties of ultrasonicated kafirin or zein, so one can only assume that because of the loss of interactions in the modified protein, the resulting doughs would be unable to form a network able to entrap gases and withstand their expansion during baking.

### 3.4. Genetic Modifications

Genetic modifications are aimed to alter the genetic material (DNA) in a way that does not occur naturally [100]. The intended changes in the DNA sequence confer modifications in the sequence of amino acids, which will bestow particular traits and properties [101]. Reproduction and natural recombination are not considered genetic modifications; the modification process must not occur naturally, as is the case of recombinant DNA technology, which involves the insertion of external DNA fragments using a vector [102].

To the knowledge of the authors, in recent years, the only work done where the modification fits this criterion is Elhassan et al.’s research with transgenic sorghum, where γ-kafirin expression was suppressed. These genetically modified seeds formed stronger doughs than null controls, as evidenced by the higher maximum force showed during compression tests (up to a 95.9% increase) [71]. The study found that the suppression of kafirin subclasses resulted in a less dense endosperm with modified protein bodies and improved protein-starch interactions through hydrogen bonding. As mentioned in Section 3.1, the complete elimination of the γ fraction of the prolamin can be detrimental to the final viscoelastic properties of bread; however, its reduction does not appear to have the same effect. This can indicate that the initial and final protein composition and structure are essential for the effectiveness of the modification, where an increase in hydrogen bonding sites would be beneficial to yield stronger dough.

## 4. Methodologies for the Measurements of the Gluten Substitute and the Gluten-Free Dough Properties

The efficiency of protein modification for gluten substitution can be assessed by studying dough rheology and thermodynamic properties. Both can be excellent predictors of the quality of finished bakery products, so their use has been reported in a wide variety of investigations [103,104]. Properties such as the viscoelastic moduli and glass transition temperature have been employed to identify the potential of modified materials as gluten substitutes. These properties are measured to confirm if the modification was successful and to be able to compare the final product with a null control. These methodologies are classified into proteins characterization, rheological evaluation, and thermal properties measurements in the following section.

### 4.1. Characterization of Protein Modifications for Gluten-Free Doughs

To show evidence of protein composition and structure before and after a modification, the characterization of materials has been done with different analytical techniques such as spectroscopy, electrophoresis, and microscopy, which complement each other.

#### 4.1.1. Fourier Transform Infrared Spectroscopy

Fourier Transform Infrared Spectroscopy (FTIR) has been used to study proteins to determine their secondary structures [105]. This technique consists of exciting the vibrational transitions of a molecule using infrared radiation to measure the light intensity absorbed by the sample, which depends on the polarity of the bonds and the intra and intermolecular forces exerted on them [106]. In the case of proteins, peptide bonds can be used as a source of information; amides present in peptide bonds signal in the infrared spectra mainly in bands I (~1650 cm^−1^) and II (~1550 cm^−1^), which have been characterized on proteins to identify secondary structure sensible regions [107]. Studies on zein and kafirin did by Taylor et al. have shown that approximately 56.7 and 49.0% of protein structures are α-helix, respectively, and changes in these structure compositions can give information on the impact of the analyzed treatments [63,73]. It is important to mention that his technique is not limited to study protein structures and is also used to determine starch changes. Raungrusmee et al. used FTIR to assess the crystallinity of rice starch in gluten-free bread by observing a decrease in the carbon-oxygen bond signal for starch, which was attributed to the discontinuities provided by the addition of hydrocolloids and bran, which significantly increased the viscous behavior [68].

#### 4.1.2. D-Electrophoresis

Another technique used in the characterization of proteins in gluten-free breadmaking is 2D-Electrophoresis. This technique consists of separating proteins in gels according to their isoelectric point under a pH gradient (first dimension) and then using an anionic detergent (dodecyl sulfate) to impart them a negative charge relative to their size so that they can segregate relative to their molecular weight (second dimension) [108,109,110]. The characterization of kafirin and zein fractions is usually done using sodium dodecyl sulfate-polyacrylamide gel electrophoresis (SDS-PAGE) using established molecular weight bands assigned to the α, β, γ, and δ sub-classes [63]. In gluten-free protein modification, this technique can evaluate the effect that a physical modification has on the size of sub-class peptides and changes in the molecular weight of protein chains. In work by Federici et al., the use of SDS-PAGE found that extrusion increased the number of small peptides of zein, while higher temperatures resulted in higher molecular weights because of disulfide bonding formation [64]. The assessment of molecular protein weights is important when it is considered that proteins have a range where they maximize their viscoelastic behavior (see Section 3.3), making this technique very useful to studies where the modification must decrease protein size and weight.

#### 4.1.3. Microscopy

One of the most popular techniques to study the surface morphology and microstructure of a prepared gluten-free bread is Scanning Electron Microscopy (SEM). In SEM, an image is obtained by scanning an electron beam over a sample and collecting the signals from the impact of the incident electrons [111]. Because of its relative simplicity of use, the technique has been applied to study several structure interactions on the surface, which include fibrous structure detection, protein network porosity, and the tendency of protein aggregation [64,65,68].

Several studies have opted to use Confocal Laser Scanning Microscopy (CLSM) as an alternative to SEM; this technique uses the emitted fluorophores of a sample after a laser scan to generate the image [112]. Taylor et al. used this technique to show the fibril formation of zein and kafirin resins, while Elhassan et al. used it to investigate the internal structure of kafirin doughs, an unavailable application for SEM [63,71]. Since both microscopies techniques can study the microstructure of a sample, the election of equipment would depend on the information needed. For surface evaluation, SEM is preferred for its high resolution (up to 5 nm) when compared to CLSM (up to 250 nm) [113]. On the other hand, CLSM can give useful information about the internal structure, which SEM is unable to view [112]. Another factor that can be helpful when considering microscopy techniques is the pretreatment of the sample, as described in Table 3, SEM uses more expensive reactants and longer treatments than CLSM.

### 4.2. Measurement of Rheological Properties of Gluten-Free Doughs

Among the most important functionality tests on gluten and gluten-free doughs are the ones that determine dough rheological properties because they are associated with processing parameters as optimum water absorption and mixing time; also, they can be used to evaluate the quality of bakery products [114].

The rheometer is used to comprehend the dough rheological effects of a modification on final products [67]. This equipment works by applying a rotational or sinusoidal (oscillatory) shear deformation on a sample and measuring the resulting stress response, which can be used to study the viscoelastic moduli (G’ and G”) of gluten-free bread [67,69,71,115]. Since both techniques obtain the same information, the election would depend on the type of material studied. Doughs are commonly studied using oscillatory modes with parallel plate geometries, whereas batters, commonly studied using a cylinder geometry with a rotational mode, as illustrated in the equipment column of Table 4 [64,65,67,69].

In rheological studies, the storage modulus (G’) measures the recovered stored energy of the material, while the loss modulus (G”) measures the dissipated energy per deformation. These parameters are also referred to as elastic and viscous moduli, respectively [116]. The former predicts how much a material will deform when stress is applied, and the latter, how fast the material will flow [117]. In breadmaking, softer doughs have less elastic behavior (lower G’), since higher G” would indicate a more viscous behavior, and hence a less ordered dough structure [71].

Other rheological properties, such as volume and strength, can be assessed using the viscoelastic moduli G*, δ, and λ (Table 4). These parameters complement critical information related to the capacity of doughs to retain gas. Tunçil et al. obtained both G values to calculate the complex modulus (G*), where lower values indicate weaker doughs [70]. Another studied parameter is the loss tangent (tan δ), obtained from dividing G” by G’. Several studies have stated that by lowering this value, an increase in the specific volume of the bread is observed since lower G” is associated with stronger dough gas retention [67]. The retention of these properties after deformation can also be measured. Federici et al. calculated the inverse of G’ before and after deformation (λ_1_ and λ_2_) and the degree of elasticity (α). The α values close to 0 and smaller differences between λ_1_ and λ_2_ is related to a product that maintains its elastic behavior, while a loss of elasticity can be attributed to quick staling [64]. Table 4 summarizes the methodologies and the properties measured with a rheometer for gluten-free substitutes evaluation.

The rheometer is not the only equipment used to analyze the viscoelastic behaviors of doughs. The texture analyzers are also effectively used because doughs undergo biaxial extension during leavening; extension tests have been applied with a texturometer to assess protein effects on dough’s structure [64]. Instead of measuring the stress response of a sample, like in the case of rheometers, texturometers generate a force-extension curve from deforming the sample until its fracture [118]. The peak force and maximum extensibility before material rupture are used to evaluate the strength of the prepared gluten-free doughs; higher values would be preferable after modification since they indicate that protein interactions were strengthened and will produce a stronger dough [64,71,73]. Repeating this test for several days can give information on the viscoelastic and retaining capability of a product. In a study by Taylor et al., when a dough showed values that exceed those of control bread, harder products were obtained and indicated staling and loss of elasticity during storage [63].

Using the same equipment for extension tests, the texture profile analysis (TPA) parameters have been calculated to grade the quality of the final product, as in the case of bread prepared by Azizi et al., Turkut et al., and Villanueva et al. [54,66,69]. In a TPA analysis, hardness represents the necessary force to deform a material, springiness is the rate at which it returns to an undeformed state, cohesiveness is the strength of the internal bonds, and chewiness is the energy required to masticate the sample [119]. In breadmaking, the effectiveness of an addition or modification must show an increase in values for springiness and cohesiveness to indicate that the product has higher stability and freshness after the treatment [66]. However, like peak force, hardness and chewiness must show intermediate values, or else the bread would be too hard or too soft for consumption.

Among other instruments used to evaluate dough rheological properties are (a) farinograph, used in the most applied methodology to evaluate rheological properties of dough, based on the determination of the optimum amount of water to achieve a consistency of 500 farinograph units (FU) and in the mixing tolerance index, calculated as a drop in the dough consistency after five minutes dough development time; (b) extensigraph, an instrument used to measure rheological properties of dough obtained with farinograph, determining the dough’s resistance and extensibility: (c) mixograph, and instrument that works with the same principle of the farinograph, but only requires 10 to 35 g sample and 7 to 8 min, instead of 50 or 300 g flour and more than 15 min in farinograph; and (d) alveograph, which measures dough properties when air is injected into a disc of dough pre-formed under specific parameters, simulating gas retention during fermentation [64,114,120,121]. This specific study of dough gas retention can also be measured using the volume of the product after fermentation and/or baking. Some examples are the use of laser technology (laser volumeter as Volscan profiler from Stable Micro System), seed displacement (rapeseed displacement, official AACC method 10-05.01), and rheofermentometer used to measure fermentation with a weight on the dough; its height and gas development is measured by height and pressure sensors [122,123,124].

### 4.3. Measurement of Thermal Properties of Gluten-Free Doughs

The thermal behavior of doughs is dictated by two central mechanisms: starch gelatinization and protein denaturation, both of which contribute to the final quality of bakery products [125]. One of the most common analytical techniques to study these endothermic processes is differential scanning calorimetry (DSC) [63,68,71,126]. In the food industry, DSC has been used to study the stability and denaturation of proteins by the characterization of their melting (T_m_) and glass transition temperatures (T_g_) and the estimation of transition enthalpy changes (ΔH) [127].

Glass transition temperature (T_g_) is the temperature above which the relative mobility of the molecules is increased, giving the material a more rubber-like behavior [128]. In gluten-free breadmaking studies, T_g_ has been used as an indicator for protein stability; in the work of Taylor et al., higher values of T_g_ were observed in the thermogram for kafirin than for zein, indicating that sorghum prolamin exhibited higher stability due to stronger bonds and interactions [63]. This comparison was related to the amount of cysteine available for both prolamins since the formation of disulfide bonds would be higher in a protein with more cysteine residues. It can be inferred that T_g_ can also give information on the tertiary structure of proteins since an increase in this value by a modification can be an indicator of the formation of covalent bonds between side chains.

Although ΔH of gelatinization is a property largely dependent on starch composition, protein interactions affect its final value, making it valuable for studying the stability of a baked product. The presence of non-starch components such as hydrocolloids and proteins decreases the susceptibility of starch to recrystallize, hence why less energy would be required, and both the temperatures for the transition and ΔH will be reduced [68,126]. For breadmaking, this decrease in enthalpy and temperature values can be helpful. Bourekoua et al. mention that the improvement of parameters assessed in optimized bread can be explained by less stable starch structures which allow earlier gelatinization and interactions between dough components as observed by Raungrusmee et al. in the case of supplementing hydrocolloid and rice bran, concluding that the elastic properties and firmness increased with the treatment [68,126]. It can be inferred that lower ΔH of dough gelatinization is related to a higher gas retention capacity and viscoelastic. A summary of the thermal properties evaluated with a DSC in modified gluten-free bread is presented in Table 5.

## 5. Future Perspectives

The importance of the study of cereal protein modification and methodologies to analyze their application in gluten substitution was presented in this review. Some advances have already been made in gluten substitution research; however, gaps of information remain in the field, which presents themselves as areas of opportunity for future studies. The following are recommendations spotted during this research.

- In some cases, studies focus on finding the best set of variables and modifications to obtain higher quality products, often pairing modification techniques to save money and resources. However, this information cannot be used to fully understand the individual effects contributed by the techniques, and separate studies should be done to elucidate the effect that a treatment can have on the final product and its protein content.
- Non-covalent interactions have been assigned for the explanation of most improvements by non-chemical/enzymatic modifications. The full mechanism is not yet fully understood; hence, the critical protein/starch systems should be studied separately to fill the information gaps and introduce factors, such as other forces, overlooked by previous authors. Computational software can be used to generate simulations that model the forces and interactions between starch and protein.
- Genetic modification is still a relatively new method for the modification of gluten substitutes, and thus more studies should be performed to improve protein properties and functionalities. Amino-acid insertion or replacement can be an alternative to better approach the properties of gluten, keeping in mind to avoid the insertion of fractions or sequences that cause the allergic response in intolerant individuals.
- Pseudocereals have been studied as gluten-free alternatives; however, they have mostly been used as additives in flours containing other protein sources. Additional studies need to be done to give a verdict on their proteins’ susceptibility to chemical and physical modifications.
- Compared to a sample made from gluten, viscoelastic parameters can estimate the potential that a gluten-free product has. The obtainment of these values for each modification can help design methodologies to improve further the quality and approximation of the final product of a complete gluten imitation. Using the obtained values, an alternative might be creating software that compiles the texture, rheological, and thermal properties of gluten-free bread to generate a score that compares it with gluten bread properties.
- Given that gas retention plays a critical role in the efficacy of a gluten substitute in dough systems, the retention capacity of gluten-free bread should be tested. The use of equipment, such as alveographs, can simulate the fermentation process during breadmaking.

## 6. Conclusions

This review presents the most recent advances published on protein modifications of cereals and pseudocereals to substitute gluten. The studies presented indicate that protein modifications help improve the final properties of gluten-free baking items by the enhancement of interactions with other ingredients. The analyzed information showed that the studied protein sources could reproduce the viscoelastic behavior of gluten after physical, chemical, enzymatic, or genetic modifications. The effectiveness of treatments depends on the type of cereal protein. For instance, maize and sorghum proteins’ high hydrophobicity needs structural changes, while rice and pseudocereals lack proper protein support, which is why non-covalent interactions need to be formed in order to produce a good substitute. The increase of covalent bonds between amino acids and non-covalent interactions with non-protein components, such as starch, can improve the gas retention capabilities of the protein matrix. Understanding prolamin interactions with their surroundings is vital for future studies that should emphasize the optimization of gluten-free products and their complete assimilation of the functional properties of gluten.

## Figures and Tables

**Table 1:** Protein content and composition of cereals and pseudocereals used in recent works for gluten substitution in bread.

Cereal/Pseudo-Cereal	Seed Protein Content	Major Storage Proteins	Predominant Amino Acid Content	References
Maize	6.0–12.0%	α, β, γ, δ-Zein	Glutamine 22.5%	Alonso-Miravalles, 2018 [52] Espinosa, 2015 [9] Geraghty, 1981 [55]
			Leucine 20.9%	
			Proline 9.6%	
			Alanine 8.8%	
			Phenylalanine 8.2%	
Rice	6.0–8.0%	12S-Globulin 11S-Globulin	Glutamine 16.7%	Amagliani, 2017 [49] Shewry, 2002 [48]
			Aspartate 8.4%	
			Arginine 8.1%	
			Leucine 7.7%	
			Valine 5.3%	
Sorghum	6.0–18.0%	α, β, γ, δ-Kafirin	Glutamine 28.2%	de Mesa-Stonestreet, 2010 [22] Espinosa, 2015 [9] Xiao, 2015 [40] Rhodes, 2017 [56]
			Leucine 17.5%	
			Alanine 11.8%	
			Proline 10.2%	
			Phenylalanine 6.6%	
Quinoa	13.8–16.5%	11S-Globulin 2S-Albumin	Glutamine 13.2%	Dakhili, 2019 [57] Navruz-Varli, 2016 [58] Janssen, 2017 [59]
			Aspartate 8.0%	
			Arginine 7.7%	
			Leucine 5.9%	
			Proline 5.5%	
Amaranth	12.0–22.0%	11S-Globulin 7S-Globulin	Glutamine 16.2%	Grundy, 2020 [60] Kumar Maurya, 2018 [61] Janssen, 2017 [59]
			Glycine 11.7%	
			Aspartate 9.0%	
			Serine 8.2%	
			Arginine 7.6%	
Buckwheat	11.0–15.0%	13S-Globulin 8S-Globulin 2S-Globulin 2S-Albumin	Glutamine 19.4%	Alonso-Miravalles, 2018 [52] Janssen, 2017 [59] Sytar, 2016 [62]
			Arginine 11.2%	
			Aspartate 9.5%	
			Proline 7.9%	
			Leucine 5.9%	

**Table 2:** Recent works on the modification of proteins for the substitution of gluten.

Cereal	Protein Modification Type	Description of Protein Modification	Results of Modification	Reference
Maize	Chemical	Defatting of zein with hexane and extraction with 70% (*w*/*w*) aqueous ethanol.	Obtainment of high α-prolamin. Increase of firmness with the presence of α-zein.	Taylor, 2018 [63]
	Physical	Extrusion of zein at 90, 120, 140, and 160 °C. Addition of corn, rice, or potato starch.	Improvement of elasticity. Higher elasticity and peak stress with rice starch.	Federici, 2020 [64]
	Physical/chemical	Microfluidization of corn gluten meal (62% zein). Addition of guar gum or hydroxypropyl methylcellulose (HPMC).	Decrease of specific volume and porosity with microfluidization. Higher elastic moduli.	Ozturk, 2018 [65]
Rice	Chemical/enzymatic	Addition of corn starch, inulin, xanthan gum, transglutaminase, protease, lipase, and quinoa flour to rice flour.	A small increase in specific volume with protease. Decrease of staling. No significant change in springiness.	Azizi, 2020 [66]
	Enzymatic	Addition of proteases (papain, Protin SD-AY, Protin SD-NY, or Newlase F) to rice flour.	Increase of specific volume except with Newlase F. Improvement of viscoelastic moduli and elastic behavior.	Honda, 2018 [67]
	Chemical	Addition of xanthan gum and inulin to rice starch and bran.	Increase of firmness. Obtainment of tensile strength and elasticity. Porous microstructure.	Raungrusmee, 2020 [68]
	Physical	Microwave radiation of 20 and 30% moisture content rice flour.	Improvement of viscoelastic moduli and specific volume. Decrease of firmness and springiness.	Villanueva, 2019 [69]
Sorghum	Enzymatic	Addition of transglutaminase to 10, 20, and 30% sorghum substituted wheat flours.	Increase of firmness and elasticity. No changes in volume.	Tunçil, 2019 [70]
	Genetic	RNA interference technology suppressed α, γ, and δ kafirin.	Higher maximum force and viscosity.	Elhassan, 2017 [71]
	Chemical	Defatting of kafirin with hexane and extraction with 70% (*w*/*w*) aqueous ethanol. Extraction of γ-kafirin with 0.05M sodium lactate containing 2% (*v*/*v*) 2-mercaptoethanol.	Obtainment of high α-prolamin. Increase in the firmness of bread and a decrease in % stress recovery with the absence of γ-kafirin.	Taylor, 2018 [63]
	Physical	Thermal treatments on sorghum flour (90 and 125 °C) during 15, 30, and 45 min.	Specific volume increases. Peak viscosity increased with time. Decrease in firmness with time.	Marston, 2017 [72]

**Table 3:** Techniques used for the study of gluten-free microstructure.

Tested Sample	Sample Pretreatment	Microscope	Microscopic Features	Reference
Genetic suppression of γ-kafirin	Addition of three drops 0.02% Acid Fuchsin dye in 1% acetic acid Heated at 60 °C 1 min	Zeiss 510 META system CLSM Excitation wavelength: 405 nm	Protein matrix density and microstructure	Elhassan, 2017 [71]
Extrusion of zein	Sputter coated with Pt Flash-frozen in liquid nitrogen at −185 °C	FEI NOVA nanoSEM Field Emission SEM	Morphology of zein Detection of fibrous microstructures.	Federici, 2020 [64]
Microfluidization of corn gluten meal	Freeze-dried 48 h Coated with Au-Pd by sputter coater device	Quanta 400F Field Emission SEM Voltage: 20 kV	Observe the aggregation tendency of zein.	Ozturk, 2018 [65]
Addition of hydrocolloids to rice bran	Coated with Au	JSM 6310F SEM Voltage: 5 kV	Observe the porosity of the microstructure.	Raungrusmee, 2020 [68]
Chemical extraction of γ-kafirin and zein	No pretreatment	Zeiss 510 META system CLSM Excitation wavelength: 488 nm	Morphology of zein and kafirin Detection of fibrous microstructures.	Taylor, 2018 [63]

**Table 4:** Methodologies used for the evaluation of gluten-free substitutes with a rheometer.

Sample Modification	Sample Pretreatment	Rheometer Characteristics	Studied Parameters	Reference
Genetic suppression of γ-kafirin (dough)	No pretreatment	Physica MCR 101 rheometer Parallel plate geometry 25 mm diameter/2 mm gap 25–150 °C at 6.25 °C/min 6.3 rad/s 0.01–100% strain rate	G’ G” tan δ Transgenic sorghum > control sorghum	Elhassan, 2017 [71]
Extrusion of zein (dough)	15 min rest at room temperature inside a polystyrene box with a water beaker	TA Instruments ARG-2 Model rheometer Parallel plate geometry 40 mm diameter Above 35 °C	α: 0.43	Federici, 2020 [64]
Protease addition to rice flour (batter)	No pretreatment	Physica MCR 301 rotational rheometer Coaxial cylinder 25 mm inner diameter/26 mm outer diameter 30–90 °C at 2.5 °C/min 10 rad/s angular velocity 0.1% strain	G’: 2160 Pa G”: 584 Pa tan δ: 0.27	Honda, 2018 [67]
Microfluidization of corn gluten meal (dough)	5 min rest at room temperature	TA Instruments AR2000ex rheometer Parallel plate geometry 20 mm diameter/2 mm gap 25 °C 6.283 rad/s 0.01–10% strain rate	G’: 140,000 Pa G”: 40,000 Pa	Ozturk, 2018 [65]
Transglutaminase addition to sorghum/wheat flour (dough)	No pretreatment	TA Instruments ARG-2 Model rheometer Parallel plate geometry 40 mm diameter 0.01–50 rad/s 0.5%	G*: 60,000 Pa δ: 18	Tunçil, 2018 [70]
Microwave radiation to rice flour (dough)	5 min rest at room temperature	Malvern Instruments Kinexus Pro+ rheometer Parallel plate geometry 40 mm diameter/1 mm gap 25 °C	G’: 3238 Pa G”: 1485 Pa tan δ: 0.49	Villanueva, 2019 [69]

**Table 5:** Methodologies used for the evaluation of thermal properties of gluten-free doughs in recent works.

Sample Modification	Sample Pretreatment	Equipment	Studied Parameter	Reference
Chemical extraction of γ-kafirin and zein	Dried in a desiccator for 14 days	DSC 25–280 °C scans Heat rate of 10 °C/min under nitrogen pressure (40 bar)	T_g_ Kafirin T_g_ > Zein T_g_	Taylor, 2018 [63]
Genetic suppression of γ-kafirin	Addition of deionized distilled water for a total weight of 36 mg	DSC 30–120 °C scans Heat rate of 10 °C/min under nitrogen at normal air pressure A flow rate of 30 mL/min	ΔH of gelatinization: 3.1 J/g	Elhassan, 2017 [71]
Addition of hydrocolloids to rice bran	No pretreatment	DSC 25–200 °C scans Heat rate of 10 °C/min Cool rate of 25 °C	ΔH of gelatinization: −6691.51 J/g	Raungrusmee, 2020 [68]

## Data Availability

Data sharing does not apply to this article.
